# Body composition parameters combined with blood biomarkers and magnetic resonance imaging predict responses to neoadjuvant chemoradiotherapy in locally advanced rectal cancer

**DOI:** 10.3389/fonc.2023.1242193

**Published:** 2023-11-22

**Authors:** Jianguo Yang, Qican Deng, Zhenzhou Chen, Yajun Chen, Zhongxue Fu

**Affiliations:** Department of Gastrointestinal Surgery, The First Affiliated Hospital of Chongqing Medical University, Chongqing, China

**Keywords:** rectal cancer, neoadjuvant chemoradiotherapy, pathological complete response, body composition parameters, blood biomarkers, magnetic resonance imaging

## Abstract

**Aim:**

To investigate whether body composition parameters combined with systemic inflammatory markers and magnetic resonance imaging (MRI) can predict the pathological complete response (pCR) following neoadjuvant chemoradiotherapy (NCRT) in locally advanced rectal cancer (LARC).

**Methods:**

A retrospective analysis of data on LARC patients treated with NCTR and radical surgery between January 2013 and May 2023 was performed. Body composition parameters were assessed by measuring the skeletal muscle index (SMI), subcutaneous adipose index (SAI), and visceral adipose index (VAI) at the third lumbar vertebra level by computed tomography (CT). Inflammatory markers such as neutrophil to lymphocyte ratio (NLR) were obtained from laboratory tests performed prior to NCRT. MRI was conducted to evaluate MRI tumor regression grading (mrTRG). Logistic regression analyses were employed to identify factors affecting the pCR. The risk score of pCR was computed by a nomogram. The discrimination of the nomogram was determined using C-index and calibration curve.

**Results:**

Two hundred and ninety-one patients with LARC were enrolled in the study, 55 (18.9%) of whom achieved pCR after NCRT. Multivariate analysis suggested that pre-NCRT NLR≥2.6 (OR=0.378, 95% CI 0.164-0.868, P=0.022), mrTRG 3-5 (OR=0.256, 95%CI 0.121-0.54, P<0.001), and pre-NCRT L-SMI (OR=0.292, 95% CI 0.097-0.883, P=0.029) were independent risk factors for pCR. ROC curves analysis demonstrated that the performance of mrTRG combined with pre-NCRT NLR and pre-NCRT L-SMI in predicting pCR was significantly improved compared with mrTRG alone (AUC: 0.763 vs. 0.667). Additionally, mrTRG 3-5 (OR=0.375, 95% CI 0.219-0.641, P<0.001) was also an independent predictor for poor tumor regression.

**Conclusion:**

The pathological complete response of neoadjuvant chemoradiotherapy in locally advanced rectal cancer can be effectively predicted by combining the body composition parameters with blood biomarkers and magnetic resonance imaging.

## Introduction

The statistics for cancer in 2022 have shown that colorectal cancer (CRC) has the third incidence and second highest mortality rate of all cancers, and its occurrence is rapidly increasing (1). Rectal cancer represents approximately 30% of all CRCs, with most being diagnosed at an already locally advanced stage (2). The standard treatment strategy for locally advanced rectal cancer (LARC) continues to be neoadjuvant chemoradiotherapy (NCRT) in combination with total mesorectal resection (TME) (3, 4). NCRT has been found to significantly improve local control of tumors, R0 resection, and sphincter-preservation rate (5). However, there are significant differences in individualized treatment responses to NCRT in LARC. Although the majority of LARC patients exhibit a pathological tumor regression response after NCRT, only 10%-30% of LARC patients achieve pathological complete response (pCR) (6). Given that tumor regression response after NCRT is closely related to the oncological outcome of patients (7, 8), predicting pCR plays a crucial role in treating LARC.

Body composition and obesity were linked with the occurrence and prognosis of cancer. Obesity was a high-risk factor for developing CRC, as well as the potential risk factor for drug resistance and oncological prognosis (9, 10). LARC patients with obesity have lower pCR and sphincter-preservation rates, and higher postoperative complications (11). Skeletal muscle, subcutaneous adipose, and visceral adipose are important components of the body, and CT has become a popular tool for assessing body composition (12). Compared to body mass index (BMI), body composition parameters are more precise in reflecting the skeletal muscle and adipose status of patients with rectal cancer (13). Low skeletal muscle has been proven to predict poor short-term and long-term clinical outcomes in patients with CRC, gastric cancer, liver cancer, bile duct cancer, and pancreatic cancer (14, 15). Low skeletal muscle also contributes to adverse effects and decreased sensitivity of LARC patients to NCRT (16). Subcutaneous adipose and visceral adipose are also important parameters that reflect the function of the body. High subcutaneous adipose and visceral adipose were independent factors influencing the tumor regression grade (TRG), postoperative complications, and recurrence in LARC (17, 18). Several meta-analyses have shown that CT-based adiposity parameters are better predictors of short-term and long-term oncological outcomes in renal clear cell carcinoma, pancreatic cancer, and gastric cancer (19–23).

Cancer-related systemic inflammation is also connected to the development, treatment sensitivity, and prognosis of many cancers, including colorectal, gastric, prostate, and breast cancers (24). The neutrophil-to-lymphocyte ratio (NLR), monocyte-to-lymphocyte ratio (MLR), systemic immune-inflammatory index (SII), and platelet-to-lymphocyte ratio (PLR) are commonly used blood markers of systemic inflammation (24). Studies have revealed that systemic inflammatory markers are not only important predictors of pathological response to NCRT in LARC but are also influential factors of disease-free survival (DFS) and overall survival (OS) (24–26).

Magnetic resonance imaging (MRI) is widely performed for pre-treatment staging and assessment of tumor regression of rectal cancer. In particular, diffusion-weighted imaging (DWI) further effectively differentiates the residual tumor cells and the level of fibrosis in the treated area after NCRT (27). A previous study revealed that MRI tumor regression grade (mrTRG) was an independent predictor of pCR, with an AUC value of 0.721. In addition, mrTRG combined with NLR, LMR, and carcinoembryonic antigen (CEA) had a significantly higher performance in predicting pCR (AUC=0.913) (28). To date, there has been a lack of research investigating the combination of body composition parameters, mrTRG, and inflammatory markers for the purpose of predicting pCR after NCRT in patients with LARC.

Consequently, the aim of this study was to assess the potential of combining body composition parameters, systemic inflammatory markers, and mrTRG as a predictive tool for pCR following NCRT in LARC patients.

## Materials and methods

### Patients

We retrospectively analyzed data from 291 patients with LARC who underwent NCTR and radial surgery at The First Hospital of Chongqing Medical University between January 2013 and May 2023. The inclusion criteria were as follows: (1) age >18 years; (2) adenocarcinoma; (3) the distance tumor from the anus <12 cm; (4) clinical T3-4 or N+ and no distant metastasis; (5) completion of NCRT and radical surgery; (6) completion of imaging (CT and MRI) and laboratory tests before NCRT and surgery. The exclusion criteria were as follows: (1) incomplete clinical data; (2) history of other malignancies; (3) recurrent rectal cancer; (4) history of pelvic radiotherapy; (5) combination with acute or chronic infections, and hematologic diseases. This study was reviewed and approved by the Ethics Committee of the First Affiliated Hospital of Chongqing Medical University and was implemented in accordance with the Helsinki Declaration. Since this study was retrospective, written informed consent was exempted.

### Neoadjuvant therapy

The treatment regimens for patients with LARC were developed by a multidisciplinary team (MDT). The radiotherapy regimens included long-course radiotherapy and short-course radiotherapy. Long-course radiotherapy was administered as 45-50Gy in 25 fractions with concurrent oral capecitabine 825 mg/m2 twice a day during radiotherapy. Short-course radiotherapy was administered as 25Gy in 5 fractions with concurrent oral capecitabine 825 mg/m2 twice a day during radiotherapy. After completion of radiotherapy, 1-3 cycles of consolidation chemotherapy were administered. The consolidation chemotherapy regimens were XELOX (Oxaliplatin 130 mg/m2, D1, Capecitabine 1000 mg/m2 twice daily, D1-D14) and XELIRI (Irinotecan 200 mg/m2, D1, Capecitabine 1000 mg/m2 twice daily, D1-D14). All patients underwent surgery according to TME principles after completion of NCRT. The tumor regression was evaluated according to the American Joint Committee on Cancer (AJCC) 8th edition classification criteria [28]. The pathological TRG (pTRG) 0-1 was defined as tumor regression (TR), while pTRG 2-3 is defined as non-tumor regression (non-TR). pCR was defined as the absence of residual tumor cells in the specimen and lymph nodes (T0N0M0).

### Body composition

**Figure 1:**
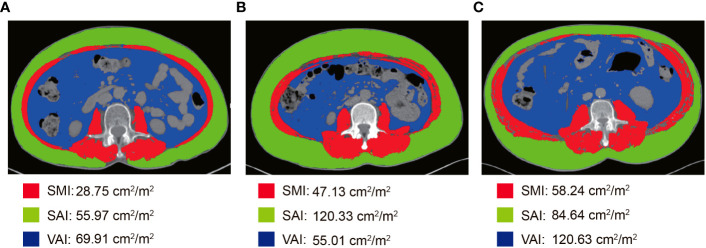
Body composition measurement based on CT images at the level of the third lumbar spine. **(A)** L-SMI; **(B)** H-SAI; **(C)** H-VAI.

All patients performed abdominal CT within 2 weeks before NCRT and surgery. Two researchers applied SliceOmatic version 5.0 (TomoVision) software to measure skeletal muscle area, subcutaneous adipose area, and visceral adipose area on CT images of cross-sections of the lumbar 3 vertebrae (L3). The Hounsfield Units (HU) range of measured tissues was as follows: skeletal muscle (-29-150 HU), visceral adipose tissue (-15-50 HU), and subcutaneous adipose tissue (-190-30 HU) (**Figure 1**) (29). The body composition area was normalized by the square of the patient’s height. We finally obtained the skeletal muscle area index (SMI), subcutaneous adipose area index (SAI), and visceral adipose area index (VAI). The change in body composition was presented as (post-NCRT-pre-NCRT)/pre-NCRT×100. The low SMI (L-SMI) was defined as the lowest sex-specific quartile cutoff value. The high SAI (H-SAI) and high VAI (H-VAI) were defined as the highest sex-specific quartile cutoff value (16). Therefore, the cut-off values for L-SMI, H-SAI, and H-VAI were 43 cm^2^/m^2^, 43.18 cm^2^/m^2^, and 59.07 cm^2^/m^2^ for males and 36.77 cm^2^/m^2^, 79.75 cm^2^/m^2^, and 49.23 cm^2^/m^2^ for females, respectively.

### Baseline hematological variables

The blood routine tests, blood biochemistry, CEA, and carbohydrate antigen 19-9 (CA19-9) were performed 1 week before NCRT. NLR = neutrophil count/lymphocyte count; PLR = platelet count/lymphocyte count; SII = (platelet count × neutrophil count)/lymphocyte count. We defined the cut-off values of NLR, PLR, and SII to maximize the discriminant power between the pCR group and the non-pCR group. Thus, the cut-off values of NLR, PLR, and SII were 2.6, 168.45, and 714.65, respectively (**Supplementary Figure 1**).

### MRI assessment of tumor regression response

The rectal high-resolution MRI was conducted within 2 weeks before NCRT and surgery. The T-stage, N-stage, tumor size, distance from the anal verge, circumferential resection margin, and extra-mural vascular invasion of rectal cancer were assessed by MRI before NCRT. The T-stage, N-stage, circumferential resection margin, extra-mural vascular invasion, and TRG of rectal cancer were assessed by MRI before surgery. Mandard TRG was used to assess MRI tumor regression response after NCRT (28). mrTRG 1-2 was defined as a good response; mrTRG 3-5 was defined as a poor response. MRI parameters were evaluated by two experienced radiologists.

### Statistical analysis

The primary endpoint of the study was pCR and the secondary endpoint was pTRG. The χ^2^ test or Fisher’s test was used for the analysis of categorical variables. Normally distributed continuous variables were expressed as mean ± standard deviation, and non-normally distributed continuous variables were expressed as median (interquartile range, IQR). The differences between the two samples of continuous variables were analyzed by Student’s t-test or Mann-Whitney U test. The Spearman correlation test was performed to compare the relationship between BMI, SMI, SAI, and VAI. Logistic regression was performed to univariate and multivariate analyses. Variables with p<0.10 in the univariate analysis were included in the multivariate analysis. The Receiver Operating Curve (ROC) was applied to predict the cut-off values of NLR, PLR, and SII. The nomogram graphs of predicting pCR were built according to the multivariate analysis. The internal validation and area under the curves (AUC) were performed to evaluate the performance of the nomogram graphs, and the C-index was used to test the discriminatory power of the nomogram graphs. P < 0.05 was considered statistically significant. SPSS 25, R version 4.1.3, and GraphPad 8 were conducted for statistical analysis.

## Results

### Basic characteristics of patients

A total of 291 LARC patients (95 female and 196 male) with a median age of 58 years fulfilled the inclusion criteria. Pre-NCRT CEA was elevated in 145 (53.26%) patients. The median of pre-NCRT NLR, PLR, and SII was 2.5 (range, 1.85-3.35), 154.07 (range, 116.91-211.48), and 574.4 (range, 389.21-870), respectively. The median of SMI, SAI, and VAI before NCRT were 45.51 cm2/m2 (range, 40.07-51.5), 36.72 cm2/m2 (range, 26.87-52.51), and 36.64 cm2/m2 (range, 19.92-55.99), respectively. Two hundred and twenty-seven (78.01%) patients with LARC suffered from long-course radiotherapy. The median interval between completion of radiotherapy and surgery was 11 weeks (range, 9-13). Anterior resection was performed in 188 patients. 55 (18.9%) patients achieved pCR after NCRT. Anastomotic leakage occurred in 27 (14.36%) patients who underwent the anterior resection procedure. Eleven (3.78%) patients underwent reoperation due to postoperative complications. Details regarding the baseline characteristics of the patients are shown in **Table 1**.

**Table 1:** The baseline characteristics.

Characteristics		Number(%)	Median(IQR)
Age,years			58 (50-65)
Sex	Male	196 (67.35%)	
	Female	95 (32.65%)	
Location	Low	146 (50.17%)	
	Middle	145 (49.83%)	
Pre-NCRT CEA	≥5ng/ml	155 (53.26%)	
Pre-NCRT CA19-9	≥27U/ml	74 (25.43%)	
Pre-NCRT NLR			2.5 (1.85-3.35)
Pre-NCRT PLR			154.07 (116.91-211.48)
Pre-NCRT SII			574.4 (389.21-870)
Pre-NCRT Albumin (g/L)			42 (39-45)
Pre-NCRT BMI (kg/m^2^)			22.77 (20.31-24.61)
Pre-NCRT SMI (cm^2^/m^2^)			45.51 (40.07-51.5)
Pre-NCRT SAI (cm^2^/m^2^)			36.72 (26.87-52.51)
Pre-NCRT VAI (cm^2^/m^2^)			36.64 (19.92-55.99)
Tumor size (cm)			5 (4.1-6.2)
Clinical T stage	T3	167 (57.39%)	
	T4	124 (42.61%)	
Clinical N stage	N0	37 (12.71%)	
	N1	79 (27.15%)	
	N2	175 (60.14%)	
Radiotherapy regimen	Short-course	64 (21.99%)	
	Long-course	227 (78.01%)	
Chemotherapy regimen	XELOX	255 (87.63%)	
	XELIRI	36 (12.37%)	
Cycle of Consolidation chemotherapy	1	41 (14.09%)	
	2	175 (60.14%)	
	3	75 (25.77%)	
mrTRG	TRG 1	25 (8.59%)	
	TRG 2	127 (43.64%)	
	TRG 3	109 (37.46%)	
	TRG 4	30 (10.31%)	
Interval between radiotherapy and surgery (weeks)			11 (9-13)
Surgical procedure	Dixon	188 (64.6%)	
	Hartmann	11 (3.78%)	
	Miles	92 (31.62%)	
ypTNM	pCR	55 (18.9%)	
	I	56 (19.24%)	
	II	105 (36.08%)	
	III	75 (25.77%)	
pTRG	TRG 0	55 (18.9%)	
	TRG 1	45 (15.46)	
	TRG 2	126 (43.3%)	
	TRG 3	65 (22.34%)	
Resection category	R0	288 (98.97%)	
Postoperation complications	Overall	84 (28.87%)	
	Anastomotic leakage	27 (14.36%)	
	Surgical site infection	53 (18.21%)	
	Ileus	24 (8.25%)	
	Hemorrhage	4 (1.37%)	
	Pulmonary infection	11 (3.78%)	
	Other	22 (7.56)	
Readmission		26 (8.93%)	
Reoperation		11 (3.78%)	

NCRT, neoadjuvant chemoradiotherapy; IQR, interquartile range; CEA, carcinoembryonic antigen; CA19-9, carbohydrate antigen 19-9; NLR, neutrophil-to-lymphocyte ratio; PLR, platelet-to-lymphocyte ratio; SII, systemic immune-inflammatory index; BMI, Body mass index; SMI, skeletal muscle area index; SAI, subcutaneous adipose area index; VAI, visceral adipose area index; mrTRG, magnetic resonance imaging tumor regression grade; pTRG, pathological tumor regression grade; pCR, pathological complete response.

### Changes in BMI and body composition parameters after NCRT

**Figure 2:**
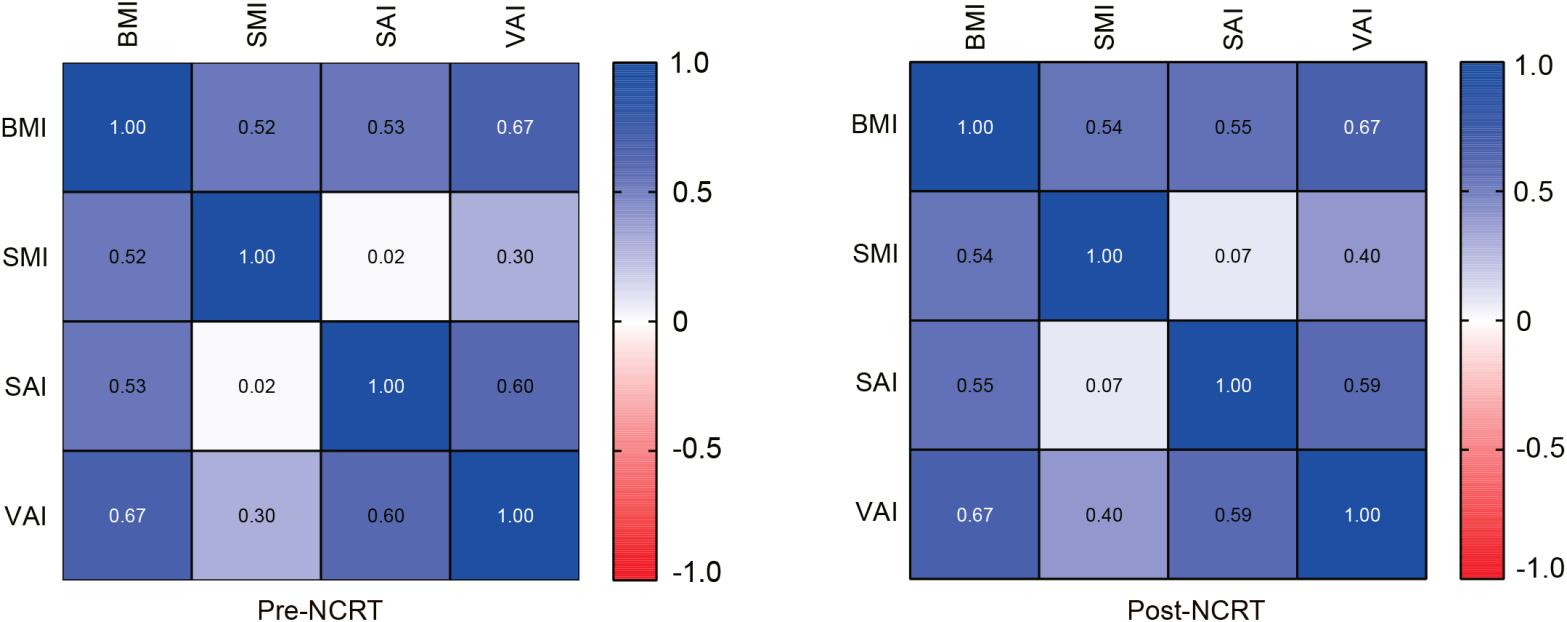
Correlation between BMI and body composition parameters before and after NCRT.

**Figure 3:**
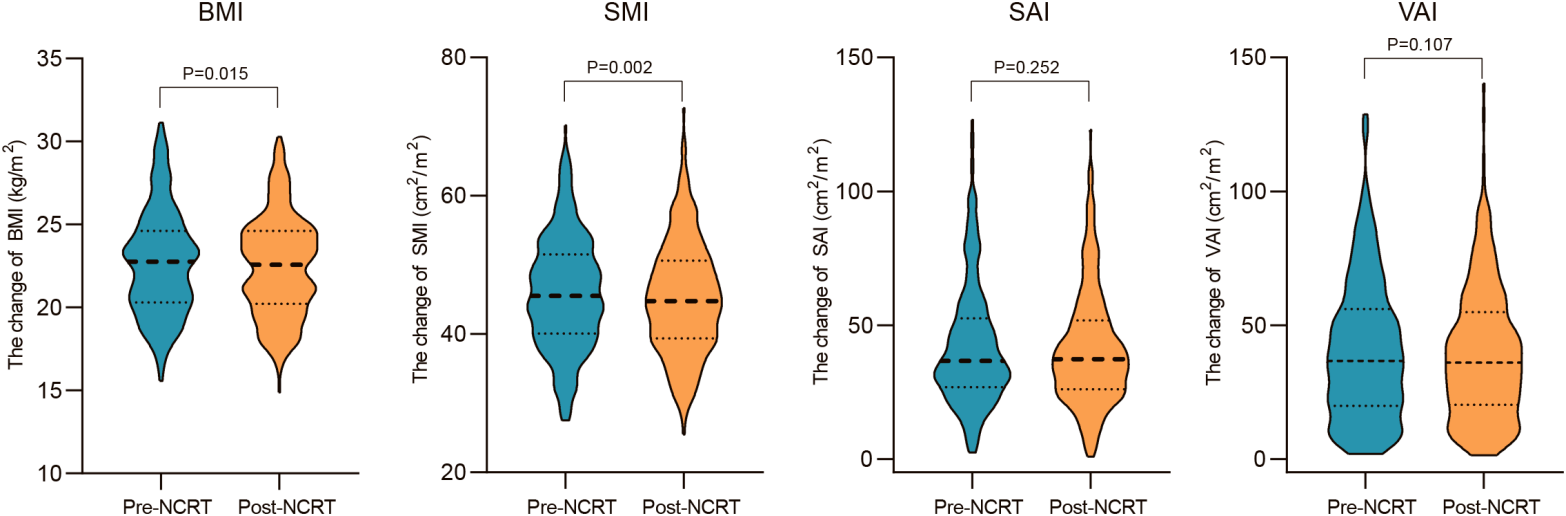
Changes in BMI and body composition parameters before and after NCRT.

Correlations between body composition parameters (SMI, SAI, and VAI) and BMI before and after NCRT were analyzed using Spearman correlation coefficients. The results showed that BMI was positively correlated with SMI, SAI, and VAI (SMI: r=0.52, P<0.001; SAI: r=0.53, p<0.001; VAI: r=0.67, P<0.001) before NCRT. There was no significant correlation between pre-NCRT VAI and SMI (r=0.02, P=0.76). The correlation between BMI, SMI, SAI, and VAI was not altered by NCRT (**Figure 2**). The median of BMI, SMI, SAI, and VAI before NCRT were 22.77 kg/m2, 45.51 cm2/m2, 36.72 cm2/m2, and 36.64 cm2/m2, respectively. The median of BMI, SMI, SAI, and VAI after NCRT were 22.58 kg/m2, 44.78 cm2/m2, 37.28 cm2/m2, and 36.06 cm2/m2, respectively. Overall, BMI and body composition parameters decreased in patients with LARC after NCRT. The post-NCRT BMI and SMI were significantly lower than pre-NCRT (P=0.015; P=0.002) (**Figure 3**). The median of changes in BMI, SMI, SAI, and VAI after NCRT were 0, -0.96%, -1.65%, and -3.04%, respectively (**Table 2**).

**Table 2:** The baseline of BMI and body composition parameters.

Variable	Pre-NCRT (IQR)	Post-NCRT (IQR)	Change of body composition (IQR)	*P* value
BMI (kg/m^2^)	22.77 (20.31-24.61)	22.58 (20.22-24.61)	0 (-4-2.31)	0.015
SMI (cm^2^/m^2^)	45.51 (40.07-51.5)	44.78 (39.36-50.61)	-0.96 (-7.15-3.84)	0.002
SAI (cm^2^/m^2^)	36.72 (26.87-52.51)	37.28 (26.09-51.76)	-1.65 (-14.79-12.5)	0.251
VAI (cm^2^/m^2^)	36.64 (19.92-55.99)	36.06 (20.30-54.84)	-3.04 (-19.13-22.21)	0.107

NCRT, Neoadjuvant chemoradiotherapy; IQR, interquartile range; BMI, Body mass index; SMI, skeletal muscle area index; SAI, subcutaneous adipose area index; VAI, visceral adipose area index.

### NCRT Baseline characteristics of patients with pCR

Fifty-five (18.9%) patients with LARC attained pCR after NCRT. The median age of the pCR group and the non-pCR group were 56 years (range, 49-66) and 59 years (range, 50.25-65) years, respectively. The proportion of female patients reaching pCR was higher than that of male patients (24.21% vs 16.33%), but the difference was not statistically significant (P=0.11). Patients with NLR < 2.6, PLR < 168.45, and SII < 714.65 before NCRT were more likely to obtain a pCR. There were no significant differences between the two groups in tumor size, clinical T stage, clinical N stage, radiotherapy regimen, chemotherapy regimen, the cycle of consolidation chemotherapy, and the interval between completion of radiotherapy and surgery. The proportion of pCR in patients with mrTRG 1-2 was significantly higher than that in patients with mrTRG 3-4 (52% vs 24.41% vs 9.17% vs 3.33%, P < 0.001). Significantly fewer patients had pre-NCRT L-SMI in the pCR group than in the non-pCR group (7.27% vs 29.34%, P < 0.001). Patients with LARC in the pCR group showed greater changes in BMI (-1.37% vs 0, P=0.021) (**Table 3**).

**Table 3:** Baseline characteristics of patients with pCR.

		pCR(n=55)	non-pCR(n=236)	P
Age, years	IQR	56 (49-66)	59 (50.25-65)	0.406^m^
Sex	Male	32	164	0.113^f^
	Female	23	72	
Pre-NCRT NLR	<2.60	43	113	<0.001^f^
	≥2.60	12	123	
Pre-NCRT PLR	<168.45	43	126	0.001^f^
	≥168.45	12	110	
Pre-NCRT SII	<714.65	47	147	0.001^f^
	≥714.65	8	89	
Pre-NCRT CEA, ng/ml	≥5	25	130	0.231^f^
Pre-NCRT CA19-9, U/ml	≥27	14	60	1^f^
Size, cm	IQR	4.8 (4-5.8)	5.1 (4.2-6.2)	0.159^m^
Clinical T stage	T3	29	138	0.453^f^
	T4	26	98	
Clinical N stage	N0	9	28	0.601
	N1	13	66	
	N2	33	142	
Clinical TNM	II	9	28	0.372^f^
	III	46	208	
Radiotherapy regimen	Short-course	12	52	1^f^
	Long-course	43	184	
Chemotherapy regimen	XELOX	51	216	1^f^
	XELIRI	4	20	
Cycle of Consolidation chemotherapy	1	4	37	0.271
	2	36	139	
	3	15	60	
Interval between radiotherapy and surgery, weeks	≤10	24	121	0.369^f^
	>10	31	115	
mrTRG	TRG 1	13	12	<0.001
	TRG 2	31	96	
	TRG 3	10	99	
	TRG 4	1	29	
Pre-NCRT BMI, kg/m^2^	>23.9	22	68	0.109^f^
Pre-NCRT L-SMI, cm^2^/m^2^		4	69	<0.001^f^
Pre-NCRT H-SFI, cm^2^/m^2^		14	59	1^f^
Pre-NCRT H-VFI, cm^2^/m^2^		17	56	0.301^f^
ΔBMI	IQR	-1.37 (-5.81-0)	0 (-3.74-3.14)	0.021^m^
ΔSMI	IQR	-2.12 (-8.03-2.14)	-0.525 (-6.83-3.9625)	0.340^m^
ΔSAI	IQR	-3.38 (-18.17-8.08)	-1.235 (-14.17-15.31)	0.221^m^
ΔVAI	IQR	-9.47 (-21.59-16.27)	-1.35 (-17.63-22.88)	0.115^m^

m, Mann-Whitney U test; f, Fisher’s test; NCRT, neoadjuvant chemoradiotherapy; IQR, interquartile range; CEA, carcinoembryonic antigen; CA19-9, carbohydrate antigen 19-9; NLR, neutrophil-to-lymphocyte ratio; PLR, platelet-to-lymphocyte ratio; SII, systemic immune-inflammatory index; BMI, Body mass index; L-SMI, low skeletal muscle area index; H-SAI, high subcutaneous adipose area index; H-VAI, high visceral adipose area index; mrTRG, magnetic resonance imaging tumor regression grade; pCR, pathological complete response; ΔBMI, The change of Body mass index; ΔSMI, The change of skeletal muscle area index; ΔSFI, The change of subcutaneous adipose area index; ΔVAI, visceral adipose area index.

### Predictors of pCR to NCRT

Univariate and multivariate analyses of LARC patients with pCR after NCRT were shown in **Table 4**. Univariate analysis indicated that pre-NCRT NLR≥2.6 (OR=0.256, 95% CI 0.129-0.511, P<0.001), pre-NCRT PLR≥168.45 (OR=0.32, 95% CI 0.16-0.637, P=0.001), pre-NCRT SII≥714.15 (OR=0.281, 95% CI 0.127-0.622, P=0.002), mrTRG 3-5(OR= 0.218, 95% CI 0.107-0.443, P<0.001) and pre-NCRT L-SMI (OR=0.19, 95% CI 0.066-0.546, P=0.002) were risk factors for pCR. Multivariate analysis was performed on variables with P<0.1 in the univariate analysis. The analysis results suggested that pre-NCRT NLR≥2.6 (OR= 0.378, 95%CI 0.164-0.868, P=0.022), mrTRG 3-5 (OR=0.256, 95%CI 0.121-0.54, P<0.001), and pre-NCRT L-SMI (OR=0.292, 95% CI 0.097-0.883, P=0.029) were independent risk factors for pCR.

**Table 4:** Univariate and multivariate analysis for pCR to NCRT.

		Univariate analysis		Multivariate analysis	
		OR (95% CI)	P	OR (95% CI)	P
Age, years	IQR	0.99(0.966-1.013)	0.386		
Sex	Male	ref	0.109		
	Female	0.611(0.334-1.117)			
Pre-NCRT NLR	<2.60	ref	<0.001	ref	0.022
	≥2.60	0.256(0.129-0.511)		0.378(0.164-0.868)	
Pre-NCRT PLR	<168.45	ref	0.001	ref	0.216
	≥168.45	0.32(0.16-0.637)		0.582(0.247-1.372)	
Pre-NCRT SII	<714.65	ref	0.002	ref	0.93
	≥714.65	0.281(0.127-0.622)		0.953(0.327-2.775)	
Pre-NCRT CEA, ng/ml	≤5	ref	0.199		
	>5	0.679(0.377-1.225)			
Pre-NCRT CA19-9, U/ml	≤27	ref	0.996		
	>27	1.002(0.511-1.965)			
Size, cm		0.94(0.782-1.13)	0.511		
Clinical T stage	T3	ref	0.438		
	T4	1.262(0.7-2.276)			
Clinical N stage	N0	ref	0.604		
	N1	0.613(0.235-1.597)	0.316		
	N2	0.723(0.312-1.677)	0.45		
Clinical TNM	II	ref	0.369		
	III	0.688(0.304-1.556)			
Radiotherapy regimen	Short-course	ref	0.972		
	Long-course	1.013(0.498-2.06)			
Chemotherapy regimen	XELOX	ref	0.771		
	XELIRI	0.847(0.277-2.586)			
Cycle of Consolidation chemotherapy	1	ref	0.289		
	2	2.396(0.802-7.16)	0.118		
	3	2.312(0.713-7.5)	0.163		
Interval between radiotherapy and surgery, weeks	≤10	ref	0.309		
	>10	1.359(0.753-2.454)			
mrTRG	TRG 1-2	ref	<0.001	ref	<0.001
	TRG 3-5	0.218(0.107-0.443)		0.256(0.121-0.54)	
Pre-NCRT BMI, kg/m^2^	>23.9	1.647(0.896-3.027)	0.108		
Pre-NCRT L-SMI, cm^2^/m^2^		0.19(0.066-0.546)	0.002	0.292(0.097-0.883)	0.029
Pre-NCRT H-SFI, cm^2^/m^2^		1.024(0.522-2.011)	0.944		
Pre-NCRT H-VFI, cm^2^/m^2^		1.438(0.754-2.743)	0.27		
ΔBMI	IQR	0.954(0.909-1.001)	0.054	0.947(0.897-1.001)	0.053
ΔSMI	IQR	1.033(0.996-1.072)	0.082	1.027(0.987-1.069)	0.185
ΔSFI	IQR	0.996(0.986-1.005)	0.373		
ΔVFI	IQR	0.994(0.986-1.002)	0.118		

NCRT, neoadjuvant chemoradiotherapy; IQR, interquartile range; CEA, carcinoembryonic antigen; CA19-9, carbohydrate antigen 19-9; NLR, neutrophil-to-lymphocyte ratio; PLR, platelet-to-lymphocyte ratio; SII, systemic immune-inflammatory index; BMI, Body mass index; L-SMI, low skeletal muscle area index; H-SAI, high subcutaneous adipose area index; H-VAI, high visceral adipose area index; mrTRG, magnetic resonance imaging tumor regression grade; pCR, pathological complete response; ΔBMI, The change of Body mass index; ΔSMI, The change of skeletal muscle area index; ΔSFI, The change of subcutaneous adipose area index; ΔVAI, visceral adipose area index.

**Figure 4:**
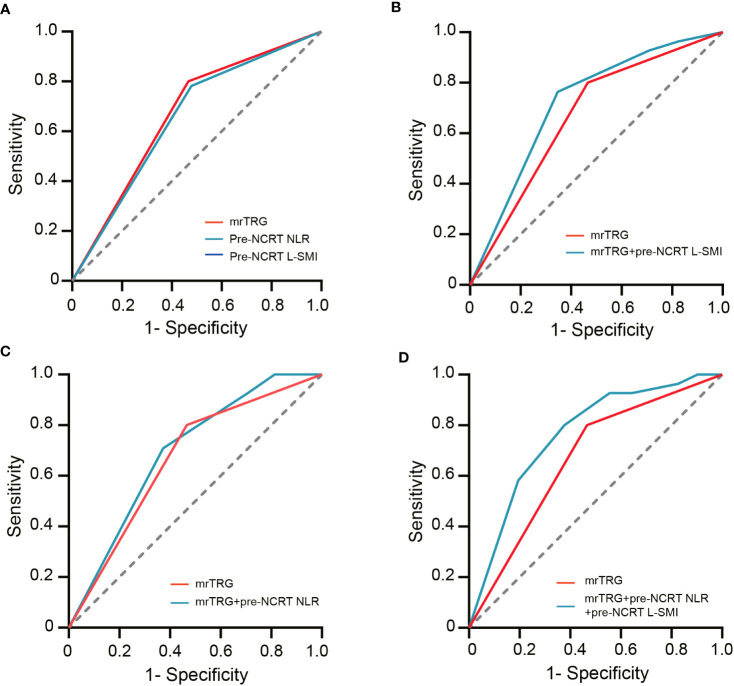
The ROC curves of assessing pCR. **(A)** the ROC curves of mrTRG, pre-NCRT NLR and pre-NCRT L-SMI alone; **(B)** the ROC curves of mrTRG + pre-NCRT L-SMI; **(C)** the ROC curves of mrTRG + pre-NCRT NLR; **(D)** the ROC curves of mrTRG+ pre-NCRT L-SMI + pre-NCRT NLR.

ROC curves were used to evaluate the performance of NLR, mrTRG, and L-SMI in predicting pCR. The results demonstrated that the AUC for pre-NCRT NLR, mrTRG, and pre-NCRT L-SMI was 0.667 (95% CI 0.592-0.742, P<0.001), 0.652 (95% CI 0.575-0.728, P<0.001) and 0.61 (95% CI 0.535-0.685, P=0.011), respectively. The performance of mrTRG combined with pre-NCRT NLR and pre-NCRT L-SMI in predicting pCR was significantly improved compared with mrTRG alone (AUC: 0.763 vs. 0.667) (**Figure 4**, **Table 5**).

**Table 5:** The AUC value of ROC curves.

Parameters	pCR	
	AUC (95% CI)	P Value
mrTRG	0.667 (0.592-0.742)	<0.001
Pre-NCRT NLR	0.652 (0.575-0.728)	<0.001
Pre-NCRT L-SMI	0.61 (0.535-0.685)	0.011
Pre-NCRT NLR+ pre-NCRT L-SMI	0.695 (0.625-0.764)	<0.001
mrTRG+ pre-NCRT NLR	0.739 (0.671-0.808)	<0.001
mrTRG+ pre-NCRT L-SMI	0.72 (0.65-0.79)	<0.001
mrTRG+ pre-NCRT NLR+ pre-NCRT L-SMI	0.763 (0.698-0.829)	<0.001

NCRT, neoadjuvant chemoradiotherapy; NLR, neutrophil-to-lymphocyte ratio; L-SMI, low skeletal muscle area index; mrTRG, magnetic resonance imaging tumor regression grade; pCR, pathological complete response; AUC, the area under the curve; ROC, receiver operating characteristic.

**Figure 5:**
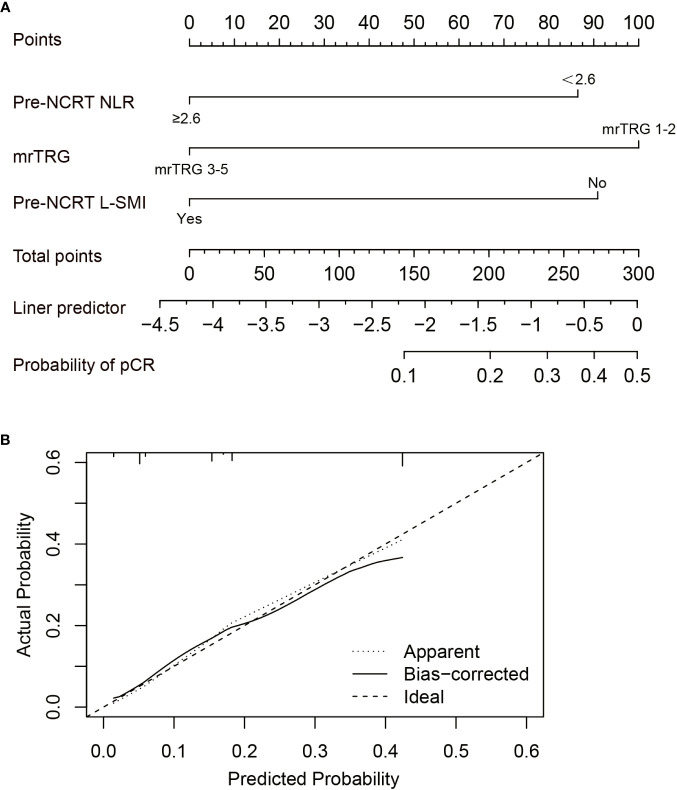
Construction of the factors for pCR to NCRT. **(A)** The Nomogram of predicting pCR; **(B)** The curves of internal validation for the nomogram.

Based on the results of multivariate analysis, pre-NCRT NLR, mrTRG, and pre-NCRT L-SMI were performed to construct a predictive nomogram for pCR after NCRT for LARC (**Figure 5A**). The probability of pCR prediction after NCRT for LARC patients can be obtained by summing the scores corresponding to pre-NCRT NLR, mrTRG, and pre-NCRT L-SMI, and then plotting a straight line to obtain the probability of achieving pCR. Patients with higher total points were more likely to reach pCR. The model was validated internally and a correction curve was drawn. The validated results showed that the predicted probability of pCR was in good agreement with the actual probability (**Figure 5B**). The discriminant ability of pCR prediction models was evaluated by the C-index. The results revealed that the C-index of the nomogram was 0.763 (95% CI 0.700-0.826).

### Predictors of tumor regression response to NCRT

pTRG 0-1 was defined as tumor regression (TR), while pTRG 2-3 is defined as non-tumor regression (non-TR). Univariate analysis showed that pre-NCRT NLR≥2.6 (OR= 0.523, 95% CI 0.318-0.859, P=0.011), pre-NCRT PLR≥168.45 (OR= 0.461, 95% CI 0.276-0.771, P=0.011), pre-NCRT SII≥714.15 (OR= 0.402, 95%CI 0.229-0.705, P=0.001), and mrTRG 3-5 (OR= 0.336, 95%CI 0.201-0.563, P<0.001) were risk factors for TR. We then conducted multivariate analysis on variables with P<0.1 in univariate analysis. The results indicated that mrTRG 3-5 (OR=0.375, 95% CI 0.219-0.641, P<0.001) was an independent predictor for non-TR (**Table 6**).

**Table 6:** Univariate and multivariate analysis for TR to NCRT.

		Univariate analysis		Multivariate analysis	
		OR (95% CI)	P	OR (95% CI)	P
Age, years	IQR	0.983 (0.963-1.003)	0.097	0.985 (0.964-1.008)	0.196
Sex	Male	ref	0.378		
	Female	0.795 (0.477-1.324)			
Pre-NCRT NLR	<2.60	ref	0.011	ref	0.471
	≥2.60	0.523 (0.318-0.859)		0.796 (0.428-1.48)	
Pre-NCRT PLR	<168.45	ref	0.003	ref	0.12
	≥168.45	0.461 (0.276-0.771)		0.606 (0.323-1.139)	
Pre-NCRT SII	<714.65	ref	0.001	ref	0.302
	≥714.65	0.402 (0.229-0.705)		0.672 (0.317-1.428)	
Pre-NCRT CEA, ng/ml	≤5	ref	0.856		
	>5	0.956 (0.589-1.553)			
Pre-NCRT CA19-9, U/ml	≤27	ref	0.685		
	>27	0.891 (0.508-1.561)			
Size, cm		0.976 (0.841-1.131)	0.743		
Clinical T stage	T3	ref	0.398		
	T4	1.234 (0.758-2.01)			
Clinical N stage	N0	ref	0.657		
	N1	0.717 (0.316-1.627)	0.426		
	N2	0.901 (0.433-1.876)	0.781		
Clinical TNM	II	ref	0.634		
	III	0.841 (0.412-1.716)			
Radiotherapy regimen	Short-course	ref	0.553		
	Long-course	1.197 (0.661-2.17)			
Chemotherapy regimen	XELOX	ref	0.912		
	XELIRI	0.951 (0.392-2.305)			
Cycle of Consolidation chemotherapy	1	ref	0.192	ref	0.211
	2	2.101 (0.944-4.679)	0.069	2.068 (0.898-4.767)	0.088
	3	1.887 (0.783-4.545)	0.157	1.621 (0.642-4.079)	0.307
Interval between radiotherapy and surgery, weeks	≤10	ref	0.838		
	>10	1.052 (0.648-1.706)			
mrTRG	TRG 1-2	ref	*<*0.001	ref	
	TRG 3-5	0.336 (0.201-0.563)		0.375 (0.219-0.641)	*<*0.001
Pre-NCRT BMI, kg/m^2^	>23.9	0.805 (0.48-1.352)	0.412		
Pre-NCRT L-SMI, cm^2^/m^2^		0.651 (0.363-1.167)	0.149		
Pre-NCRT H-SFI, cm^2^/m^2^		1.292 (0.729-2.287)	0.38		
Pre-NCRT H-VFI, cm^2^/m^2^		0.733 (0.424-1.267)	0.266		
ΔBMI	IQR	0.802 (0.957-1.035)	0.802		
ΔSMI	IQR	1.017 (0.986-1.049)	0.279		
ΔSFI	IQR	0.999 (0.992-1.007)	0.867		
ΔVFI	IQR	0.997 (0.991-1.003)	0.323		

NCRT, neoadjuvant chemoradiotherapy; TR, tumor regression; IQR, interquartile range; CEA, carcinoembryonic antigen; CA19-9, carbohydrate antigen 19-9; NLR, neutrophil-to-lymphocyte ratio; PLR, platelet-to-lymphocyte ratio; SII, systemic immune-inflammatory index; BMI, Body mass index; L-SMI, low skeletal muscle area index; H-SAI, high subcutaneous adipose area index; H-VAI, high visceral adipose area index; mrTRG, magnetic resonance imaging tumor regression grade; pCR, pathological complete response; ΔBMI, The change of Body mass index; ΔSMI, The change of skeletal muscle area index; ΔSFI, The change of subcutaneous adipose area index; ΔVAI, visceral adipose area index.

## Discussion

The pCR after NCRT is a crucial predictor of favorable prognosis in LARC. Several studies have reported a recurrence rate of 6-17% and a 5-year OS of 87-92.9% for patients who achieved a pCR (30–32). Although NCRT followed by surgery has been shown to reduce local recurrence and improve the clinical outcomes for LARC patients, this approach comes with a significant reduction in the quality of life due to radiotherapy adverse reactions, surgical complications, and permanent stoma (33, 34). Interestingly, radical surgery has been reported to have a similar recurrence rate and OS compared to local resection in LARC patients who achieved clinical complete response (cCR) following NCRT. However, local resection is known to significantly improve quality of life in patients with rectal cancer (35). Furthermore, a “wait-and-watch” approach has also resulted in similar oncological prognosis compared to radical surgery in patients who achieved cCR (36). Several factors contribute to the likelihood of achieving a pCR in LARC. One such factor is the radiation dose, which has a significant impact on the treatment outcome. In particular, tumor response can be enhanced by employing simultaneous integrated boost (SIB) with an up dose of 55-60 Gy (37, 38). Unfortunately, there are currently no reliable markers to accurately predict pCR and cCR for LARC patients after NCRT. This study evaluated the role of body composition parameters, systemic inflammatory markers, and MRI as predicting factors affecting pCR in LARC patients. The findings revealed that L-SMI, NLR, and mrTRG were independent risk factors for achieving pCR. Moreover, mrTRG was also an independent predictor of TR.

The assessment of short-term and long-term clinical outcomes in cancer patients based on L3 cross-sectional body composition parameters is superior to BMI because it provides sex-specific information regarding the patient’s skeletal muscle and adipose tissue (39–41). Nevertheless, the cut-off value of the body composition parameter remains controversial due to population differences. The cut-off value of L-SMI in Western populations may be higher than that in Eastern populations. In Western populations, the generally accepted cut-off values for L-SMI are 52.4 cm^2^/m^2^ for men and 38.5 cm^2^/m^2^ for women (42). However, two Asian studies defined the cutoff of L-SMI as the sex-specific lowest quartile which was strongly associated with CRC prognosis (43, 44). Therefore, the sex-specific lowest quartile was also defined as the cutoff value for the body composition parameters in this study.

The effect of L-SMI on tumor regression response and prognosis of LARC patients after NCRT is still unclear. A retrospective multicenter study investigated that sarcopenia was an independent risk factor for pCR and cCR but not a predictor of TR (45). In this study, the presence of sarcopenia was assessed by CT scanning of the psoas muscle region at the L3 level which was a minor muscle and cannot imply the entire skeletal muscle level. Olmez et al. analyzed the effect of sarcopenia on the pCR of LARC and identified sarcopenia, age≥60 years, the interval between surgery and completion of radiotherapy <8 weeks, and CEA≥2.5 ng/ml as risk factors for pCR through univariate analysis. However, this study did not conduct multivariate analysis of factors affecting pCR (46). It was also observed in our study that L-SMI before NCRT was an independent risk factor for pCR, but it was not a predictor of TR. Furthermore, studies have shown that L-SMI is an independent risk factor for adverse reactions to NCRT, postoperative complications, OS, and DFS in patients with LARC (16–18). However, the underlying reasons for the association between L-SMI and poor oncological outcomes or treatment response to NCRT in LARC remain unclear. Possible explanations for this included the overwhelming distribution of hydrophilic chemotherapeutic drugs such as fluorouracil and oxaliplatin in the lean body which can cause overdose of chemotherapy drugs (47). Loss of skeletal muscle in cancer patients indirectly reflected the strong invasive potential of the tumor (48). Malnutrition was also a principal factor in muscle loss, and patients with malnutrition have impaired immune status and reduced tolerance to chemotherapy (49). Additionally, L-SMI induced the accumulation of M2 macrophages, up-regulation immune checkpoint genes and proinflammatory cytokines (IL-6, IL-10, and TGF-β), and alteration the tumor microenvironment and immune status (50).

Systemic inflammation stimulated cancer cell proliferation, metastasis, immunosuppression, and alteration of the tumor microenvironment through pro-inflammatory factors (24). Several systemic inflammatory markers NLR, PLR, SII, and LMR have been demonstrated to be associated with tumor regression response and prognosis in a variety of cancers (51). A multicenter retrospective study involving 808 patients with LARC indicated that NLR>1.2 and SII>500 were independent risk factors for pCR (52). Furthermore, Liu et al. also confirmed that NLR (AUC=0.794, P=0.024) and PLR (AUC=0.740, P=0.006) were critical predictors of pCR in LARC (53). Sun et al. revealed that low NLR was an independent predictor of TR to NCRT in rectal mucinous adenocarcinoma (OR=4.025, P=0.028), but not SII (54). Our study also suggested that high NLR before NCRT can act as an independent risk factor predictor for pCR in patients with LARC. Although pre-NCRT SII and PLR were not confirmed to be independent risk factors of pCR, in a univariate analysis high SII and PLR were less likely to achieve pCR. However, the ability of systemic inflammatory markers to predict pCR, OS, and DFS in LARC remains controversial. A multicenter study indicated that NLR and PLR were neither risk factors of OS and DFS in LARC patients nor predictors of pCR and TR (26). AN et al. showed that NLR< 2.8 and PLR< 300 were not associated with pCR and 5-year OS, but PLR could be a predictor for 5-year DFS (55). Currently, there was no unified cut-off value of inflammatory markers such as NLR, PLR, and SII to predict pCR and prognosis. The cut-off value of NLR generally between 2-3 can prognosticate the pCR and outcomes (56). In this study, we predicted the optimal cut-off values for obtaining pCR by ROC curves for NLR, PLR, and SII, and ultimately indicated that NLR ≥2.6 was an independent predictor of pCR (AUC= 0.652, P<0.001).

MRI has routinely been applied for staging and treatment response of rectal cancer. Compared with conventional MRI, DWI was more effective in assessing TRG after NCRT for LARC (57). The assessment of mrTRG was also influenced by the radiologist and MRI parameters. Presently, the competence of MRI alone in predicting pCR is still unsatisfactory. Yoo et al. suggested that combination of mrTRG and blood biomarker CEA could better identify the TR to NCRT (AUC: 0.68 vs 0.728) (58). Shi et al. revealed that the proportion of pCR in patients with mrTRG 1-2 was significantly higher than that in patients with mrTRG 3-5 (70% vs 23.1%, P=0.001), and mrTRG could also serve as an independent predictor of pCR (OR=0.074 95% CI 0.011-0.499; P = 0.007). Besides, compared with mrTRG alone, the efficacy of mrTRG combined with NLR in predicting pCR was significantly improved (28). Consistent with this study, we also indicated that mrTRG 1-2 was also an independent predictor of pCR and TR after NCRT for LARC. The performance of mrTRG combined with pre-NCRT NLR and pre-NCRT L-SMI in predicting pCR was greater than that of mrTRG alone (AUC: 0.667 vs 0.763).

Despite the encouraging results observed in this study, we must consider several limitations. Firstly, this study was a single-center retrospective study that may be subject to selection bias and information bias. Secondly, the effect of mrTRG, pre-NCRT NLR, and pre-NCRT L-SMI on OS and DFS in LARC was unclear due to insufficient follow-up time. Thirdly, no uniform cut-off value of systemic inflammatory markers was performed to predict pCR and prognosis, and they were affected by a variety of factors. Finally, the cutoff value of body composition parameters in Asians is unclear, and the sex-specific quartile was conducted as the cutoff value in this study. Therefore, further research is needed to confirm that the selection method of this cut-off value is applicable to the Asian population.

In conclusion, this study demonstrated that MRI tumor regression grading combined with neutrophil-to-lymphocyte ratio and skeletal muscle index can effectively predict the pathological complete response to neoadjuvant chemoradiotherapy in locally advanced rectal cancer. mrTRG was also an independent predictor of tumor regression.

## Data availability statement

The original contributions presented in the study are included in the article/**Supplementary Material**. Further inquiries can be directed to the corresponding author/s.

## Ethics statement

The studies involving humans were approved by Ethics Committee of the First Affiliated Hospital of Chongqing Medical University. The studies were conducted in accordance with the local legislation and institutional requirements. The participants provided their written informed consent to participate in this study.

## Author contributions

YJ: Research conception, data collection, data analysis, and manuscript writing. QC: Data collection and analysis. CZ: Data collection and literature search. YC: Literature retrieval and data extraction. ZF: conception, supervision, review, editing.
