# Supplementary material for: Body composition parameters combined with blood biomarkers and magnetic resonance imaging predict responses to neoadjuvant chemoradiotherapy in locally advanced rectal cancer
Source: Front Oncol. 2023 Nov 22;13:1242193. doi: 10.3389/fonc.2023.1242193 (PMC10699861; doi:10.3389/fonc.2023.1242193)
Supplement: Supplementary file 1 [file DataSheet_1.docx]

Supplementary Material

Body composition parameters combined with blood biomarkers and magnetic resonance imaging predict responses to neoadjuvant chemoradiotherapy in locally advanced rectal cancer

Jianguo Yang^1^, Qican Deng^1^, Zhenzhou Chen^1^, Yajun Chen^1^, Zhongxue Fu^1*^

^1^ Department of Gastrointestinal Surgery, The First Affiliated Hospital of Chongqing Medical University, Chongqing, China.

*** Correspondence:** Zhongxue Fu, Email: [fzx19990521@126.com](mailto:fzx19990521@126.com)

# Supplementary Figures


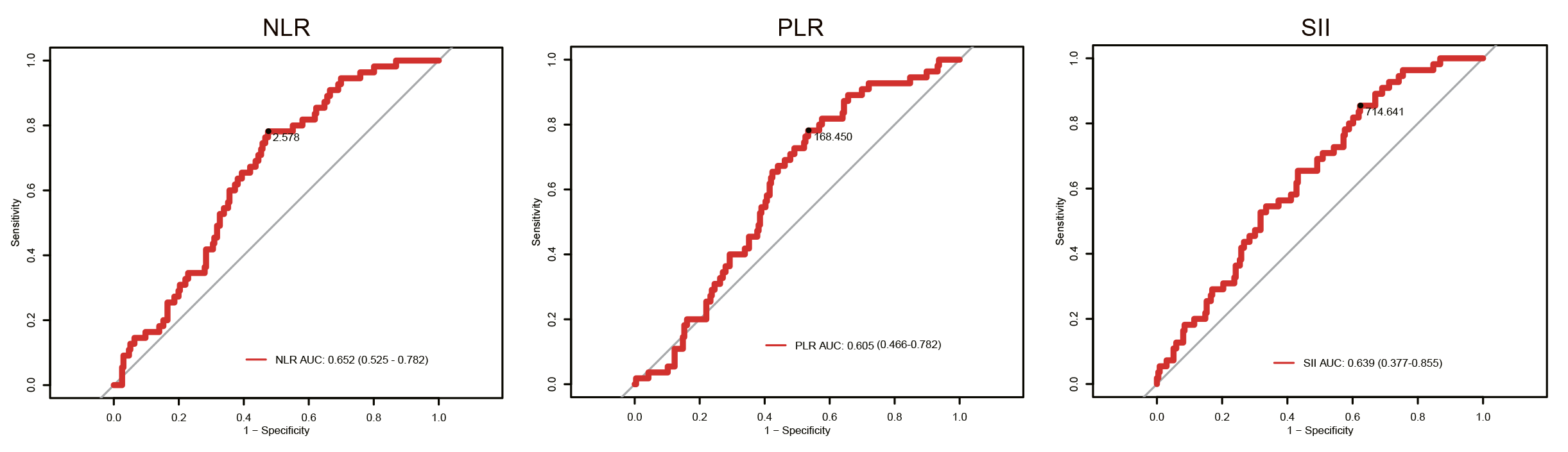


**Supplementary Figure 1.** ROC curves of NLR, PLR, and SII.
